# Metabolite components and nutritional composition of the endosperm in seven species from *Gleditsia*

**DOI:** 10.1016/j.fochx.2024.101340

**Published:** 2024-04-04

**Authors:** Feng Xiao, Yang Zhao, Xiurong Wang, Xueyan Jian, Fuhua Liu

**Affiliations:** a Institute for Forest Resources and Environment of Guizhou, Key Laboratory of Forest Cultivation in Plateau Mountain of Guizhou Province, College of Forestry, Guizhou University, Guiyang 550025, Guizhou, China; b College of Continuing Education, Yanbian University, Yanji 133002, Jilin, China

**Keywords:** *Gleditsia sinensis*, Endosperm, Metabolite components, Nutritional composition

## Abstract

As an important agricultural product, the endosperm portion of *Gleditsia sinensis* seeds, called “zào jiǎo mǐ” (ZJM) in Chinese, has gradually gained popularity and has been accepted by the public. However, there is limited information on the nutritional value and metabolic components of endosperm among *Gleditsia*. This study compared the endosperm composition among seven species. The types of metabolites, content of nutrients and amino acids were determined. A total of 4495 types of metabolites were detected. Galactose metabolism (gmx00052) was enriched in all combinations compared with *G. sinensis*. The polysaccharides content ranged from 51.49 to 80.37 g/100 g. Based on considerations of growth rate, seed yield, amino acid content, and interspecific differences, *G. fera* could be an alternative planting option to *G. sinensis*. These results can provide a reference for growers in selecting *Gleditsia* varieties and provide insights into the industrial applications of *Gleditsia* endosperm products.

## Highlights

• A total of 4495 types of metabolites were detected in all endosperm samples in *Gleditsia* genus.
• *G. fera* could be a viable alternative planting option for *G. sinensis.*
• Most of the amino acid contents of *G. fera* were higher than those of other species.

## 1 Introduction

Plants in the genus *Gleditsia*, mainly distributed in central and Southeast Asia and North and South America, have been used as sources of local and traditional medicines in many regions, especially in China (Zhang et al., 2016). There are six species, one of which comes in two varieties, of *Gleditsia* plants native to China—*G. sinensis*, *G. australis*, *G. fera*, *G. japonica*, *G. microphylla*, *G. japonica* var. *delavayi*, and *G. japonica* var. *velutina*—and an additional species (*G. triacanthos*) was introduced in the 1990s (Wanchun, Cuiling, & Yanping, 2003). *G. sinensis* (Fam.: *Leguminosae*; Gen.: *Gleditsia*) is a deciduous tree or shrub-like plant that is resistant to drought, cold, and pollution; has strong stress resistance; and is one of the first tree species used when returning farmland to forest (Liu, Wang, Zhao, & He, 2022; Liu, Yang, Wang, & Zhao, 2023). The pod of *G. japonica* and *G. delavayi* are flat and irregularly twisted; In terms of cultivation and utilization, *G. sinensis* and *G. delavayi* are primarily grown and utilized in the southern regions of China, while *G. sinensis* and *G. japonica* are predominant in the northern regions. *G. microphylla* is a shrubs or small tree, it is also frequently used as a rootstock for grafting *G. sinensis* (Han, 2014). *G. australis* seed implantation site is obviously swollen. The pod of *G. velutina* is densely yellowish green velutinous and is is a rare and endangered plant endemic to China (Yi, Junsheng, & Yonghong, 2023). *G. fera* has been subjected to artificial selection and is currently available as a cultivar with a fast-growing phenotype (Xiao, Zhao, Wang, & Jian, 2023a).

The pods, seeds, and thorns of *G. sinensis* are the main components with economic value. The seeds' endosperm is edible, while *G. sinensis* thorns, with anti-cancer and anti-inflammatory activities, are used as a medicine (Li & Ye, 2020), and a detergent can be extracted from the pods. The thorns and pods of *G. sinensis* have been studied, and their main chemical constituents are terpenoids, flavonoids, phenolic acids, and steroids (Wang, Tang, Li, & Zhou, 2008). >60 compounds have been isolated and elucidated from the genus *Gleditsia*, including triterpenes, sterols, flavonoids, phenols, and alkaloids; triterpenoid saponins are the most typical components of *Gleditsia* (found not only in the pods but also in the thorns) and may be a chemical taxonomic marker for the genus (Yu et al., 2019; Zhang et al., 2016). *G. sinensis* seeds are rich in pectin and protein components, which are used as thickeners, stabilizers, binders, gelling agents, etc. *G. sinensis* seeds can be used to make an industrial gum; this gum's structure and function are similar to those of guar gum because galactomannan is present in the endosperm of *G. sinensis* seeds as a storage polysaccharide (Kim et al., 2016). Ginsenoside polysaccharide is a neutral galactomannan (Jiang, Jian, Cristhian, Zhang, & Sun, 2011). The endosperm accounts for 37.8% of the seed composition, and the glycan content in the endosperm is 68.6% (Jianxin, Xinnan, Liwei, & Weiming, 2003).

*G. sinensis* is widely planted in plantation areas to obtain its endosperm, which is called ‘zào jiǎo mǐ’ (ZJM) and is popular among the general public due to its taste and effects. Although there are some species within the *Gleditsia* genus, whether there are other high nutrition, suitable and fast-growing varieties that can replace *G. sinensis* to mainly produce endosperm is still unknown. There is currently a lack of research on the metabolic components of the endosperm of different species in the genus *Gleditsia*. To investigate the nutritional value of endosperm and explore alternatives to *G. sinensis* as a source of endosperm, Liquid chromatography-mass spectrometry/mass spectrometry (LC-MS/MS) was used to analyze the nutritional compositions of endosperm from seven *Gleditsia* species, including the levels of crude protein, crude fat, crude fiber, ascorbic acid, minerals, and metabolites. Our study provides basic data on the nutritional composition and metabolic differences in *Gleditsia* endosperm across species, which could be useful for growers in developing and using new varieties and sources of endosperm.

## 2 Materials and methods

### 2.1 Experimental material

Pods from various species of *Gleditsia* in China were collected, including *G. sinensis* (Guiyang city, Guizhou province), *G. australis* (Conghua district, Guangdong province), *G. fera* (Ceheng city, Guizhou province), *G. japonica* (Xinmin city, Liaoning province), *G. microphylla* (Nanyang city, Henan province), *G. delavayi* (Xinyi city, Guizhou province), and *G. velutina* (Changsha city, Hunan province). The harvesting period was from 9:00 AM to 11:00 AM between November 10th and 15th, 2021. During this phase, the pods of all species had ripened. The pods exhibited a black or red color (unripe pods are green). All voucher specimens were deposited at the College of Forestry of Guizhou University (voucher ID numbers: GS202112S1 for *G. sinensis*, GA202112S1 for *G. australis*, GD202112S1 for *G. delavayi*, GF202112S1 for *G. fera*, GJ202112S1 for *G. japonica*, GM202112S1 for *G. microphylla*, and GV202112S1 for *G. velutina*).

*Gleditsia* seeds have a water-impermeable seed coat (Zhu, Dai, Ma, & Li, 2021); after harvesting the different pods, the shells and seed hulls were manually removed to obtain the endosperms. Three samples of each species were randomly selected and labeled. Three biological replicates were created. All samples were wrapped in aluminum foil and quickly frozen in liquid nitrogen, and then transferred to a freezer at −80 °C for storage.

### 2.2 Extraction and analysis of metabolites

An accurately weighed 80 mg sample was mixed with 20 μL of internal standard (L-2-chlorophenylalanine, 0.3 mg/mL; methanol configuration) and dissolved in 1 mL of 70% methanol aqueous solution. Steel balls were added to the sample, which was then placed at −20 °C for 2 min to pre-cool before grinding (60 Hz, 2 min); this was followed by ultrasonic extraction in an ice water bath for 30 min and storage at −20 °C overnight. The sample was then centrifuged for 10 min (13,000 rpm, 4 °C), and the supernatant was aspirated and filtered through a 0.22 μm organic-phase pinhole filter. The filtered product was then transferred to a sample vial and stored at −80 °C until LC–MS analysis was performed. In order to evaluate the stability of the system's mass spectrometry platform during the entire experiment, all samples were taken and mixed in equal amounts as QC samples.

The analysis conditions are described below. Chromatographic conditions: column, ACQUITY UPLC HSS T3 (100 mm × 2.1 mm, 1.8 μm); column temperature, 45 °C; mobile phase, A—water (containing 0.1% formic acid), B—acetonitrile (containing 0.1% formic acid); flow rate, 0.35 mL/min; injection volume, 2 μL. Mass spectrometry conditions: ion source, ESI; sample mass spectrometry signals were collected using positive and negative ion scanning modes. The acquired LC–MS raw data were analyzed using Progenesis QI software (Waters Corporation，Milford, USA) with the following parameters: precursor tolerance, 5 ppm; fragment tolerance, 10 ppm; retention time (RT) tolerance, 0.02 min. Internal standard detection parameters were deselected for peak RT alignment, isotopic peaks were excluded for analysis, the noise elimination level was set at 10.00, and the minimum intensity was set to 15% of the base peak intensity. An Excel file was obtained with three datasets including the *m*/*z*, peak RT, and peak intensities; RT–m/z pairs were used as the identifier for each ion. The resulting matrix was further reduced by removing any peaks with missing values (ion intensity = 0) in >50% of samples (Wang et al., 2021). An internal standard was used for data QC (reproducibility). Metabolites were identified by using Progenesis QI (Waters Corporation, Milford, USA) data processing software. Principal component analysis (PCA) was performed in order to understand the overall differences and variability between samples in each group. Differentially expressed metabolites between different treatments were evaluated using the variable importance in projection (VIP) from the OPLS-DA model. The screening criteria for differential metabolites were a VIP value of >1 for the first principal component of the OPLS-DA model and a *p-value* of ≤0.05.

### 2.3 Determination of the nutrient composition

Crude protein (CP) was determined using the Kjeldahl method (ISO, B., 2007), and the digested mixture was analyzed using an automatic Kjeldahl analyzer (K1160, Hanon, Shandong, China). Crude fat (CF), crude fiber (CFI), and polysaccharides (POL) were determined according to the Chinese national standards GB/T 5009.6–2003, GB/T 5009.10–2003, and GB/T 35818–2018, respectively. The ascorbic acid content (Vc) was determined using a 2,6-dichloro-indophenol titration method based on the Chinese national standard GB 5009.86–2016 (Zhang et al., 2020). Fatty acid content (FAC) content was determined by the refers to the previous research (Yushan, Zhanchao, Li, & Li, 2002). The extraction of saponin (SAP) refers to previous study (Zeng et al., 2017). High-performance liquid chromatography (HPLC) was performed using Thermo U3000 HPLC equipment (Thermo Fisher Scientific Inc., Waltham, MA) to detect individual amino acids after hydrolysis (Yun et al., 2022). Referring to the method previously studied (Liu, Liu, Bi, Wu, & Zhang, 2023), an inductively coupled plasma optical emission spectrometer (ICP-OES) (iCAP 7200, Thermo Fisher Scientific, USA) was used to determine the concentrations of the mineral elements K, Na, Ca, Mg, Fe, Mn, Zn, and Cu.

### 2.4 Data analysis

R v4.2.3 software (R Core Team, R, 2013) was used for the statistical and visual data analysis. A least significant difference test (LSD) was performed to test the significance differences using the agricolae v1.3.5 R package. PCA analysis was performed using the prcomp function of the stats v3.6.2 R package.

## 3 Results

### 3.1 Differences in pod and seed traits among different *Gleditsia* species

**Fig. 1:**
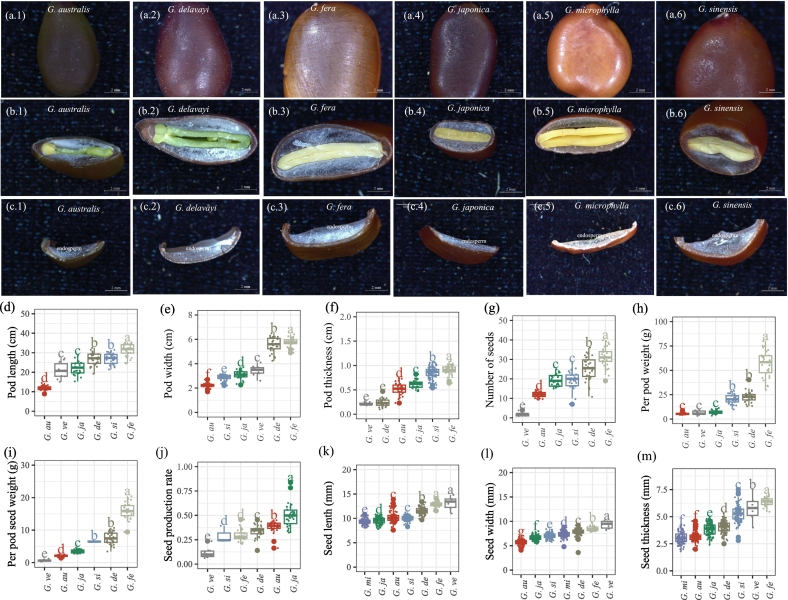
Differences in seed and pod characteristics of different *Gleditsia* species. a1-a6 were the seed of *G. australis*, *G. delavayi*, *G. fera*, *G. japonica*, *G. microphylla*, *G. sinensis*, respectively, b1-b6 were the longitudinal section of the seed of *G. australis*, *G. delavayi*, *G. fera*, *G. japonica*, *G. microphylla*, *G. sinensis*, respectively, c1-c6 were the partial endosperm of the seed of *G. australis*, *G. delavayi*, *G. fera*, *G. japonica*, *G. microphylla*, *G. sinensis*, respectively, (d) pod length, (e) pod width, (f) pod thickness, (g) number of seeds per pod, (h) single pod weight, (i) seed weight per pod, (j) seed germination rate, (k) seed length, (l) seed width, (m) seed thickness. *Note:* In d-j, the number of pods collected for *G. microphylla* was insufficient for statistical analysis; therefore, *G. microphylla* pods were excluded. *G.as*: *Gleditsia australis*; *G.de*: *Gleditsia japonica* var. *delavayi; G.fe*: *Gleditsia fera*; *G.ja*: *Gleditsia japonica*; *G.mi*: *Gleditsia microphylla*; *G.si*: *Gleditsia sinensis; G.ve*: *Gleditsia japonica* var. *velutina.*

Different *Gleditsia* species were collected (Fig. 1.a1-a6), and the seed traits were measured. The color of *Gleditsia* endosperm is milky white and gelatinous (Fig. 1.b-c). The phenotypic traits of the pods and seeds were measured, and it was found that the pods of *G. fera* were the longest (31.3 ± 3.12 cm) and widest (5.76 ± 0.33 cm) (Fig. 1.d,e); its pods were the thickest (Fig. 1.f). *G. fera* had the highest number of seeds per pod (31.37 ± 5.02), followed by *G. delavayi* and *G. sinensis* (Fig. 1.g). In terms of pod weight, *G. fera* was the heaviest (56.45 ± 13.22 g), with the total weight of seeds per pod also being the highest; the species with the next heaviest pods were *G. delavayi*, *G. sinensis, G. japonica*, *G. australis*, and *G. velutina* (Fig. 1.h,i). The order of species in terms of their seed germination rate from high to low was *G. japonica*, *G. australis*, *G. delavayi*, *G. fera*, *G. sinensis*, and *G. velutina* (Fig. 1.j). The seeds of *G. microphylla* were the shortest, while those of *G. australis* were the narrowest (Fig. 1.k,l). The seeds of *G. fera* were the thickest, while those of *G. microphylla* were the thinnest (Fig. 1.m).

### 3.2 Multivariate statistical analysis of the metabolites in the endosperm

**Fig. 2:**
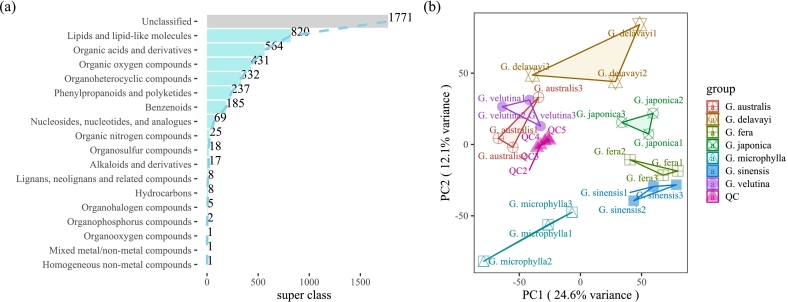
Distribution of sample metabolites and principal component analysis between samples. a: Kinds of sample metabolites; b: PCA of samples. Note: In b, different colors and shapes represent different groups.

The baseline of the LC–MS/MS chromatographic peaks was stable, and the chromatographic peaks were effectively separated. A total of 4495 types of metabolites were detected, including 820 types of lipids and lipid-like molecules, 564 types of organic acids and their derivatives, and 431 types of organic oxygen compounds (Fig. 2.a). The degree of variation within *Gleditsia* was small, among which the explanation rates of PCA_1_ and PCA_2_ were 24.6% and 12.1% (Fig. 2.b). The results from samples in the same group were relatively concentrated, indicating that the experiment had good repeatability.

### 3.3 Screening of differential metabolites and KEGG enrichment

**Fig. 3:**
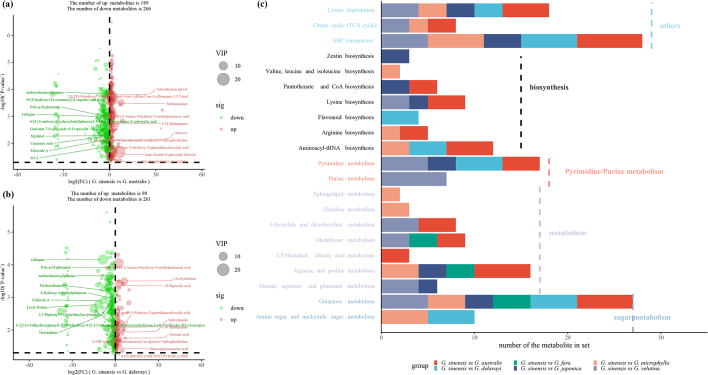
Differential metabolites and KEGG pathway enrichment. a: Volcano map of differential metabolites between *G. sinensis* and *G. australis*; b: Volcano map of differential metabolites between *G. sinensis* and *G. delavayi*; c: KEGG pathway enrichment of the differential metabolites between groups. Note: In a,b, blue represents down-regulation, red represents up-regulation. (For interpretation of the references to color in this figure legend, the reader is referred to the web version of this article.)

The VIP values of the OPLS-DA model and the results of the *t*-test were used to screen for differential metabolites between different groups in *Gleditsia*. Taking *G. sinensis* as a control, *G. australis* had the most differential metabolites with 455 kinds, of which 189 were up-regulated metabolites and 266 were down-regulated (Fig. 3.a). *G. delavayi* had 351 kinds of differential metabolites (Fig. 3.b). G. *fera* had the fewest, with 188 kinds, among which 112 were up-regulated metabolites and 76 were down-regulated. *G. japonica* had 210 types of differential metabolites, *G. microphylla* had 280 types of differential metabolites, and *G. velutina* had 386 types of differential metabolites. Regarding the common and unique characteristics of differential metabolites across the different groups, it was found that the combination of *G. sinensis* with *G. australis* had 110 unique metabolic products, while there were 51 unique metabolic products in the combination of *G. sinensis* and *G. microphylla*. The KEGG enrichment analysis of differential metabolites showed that C5-branched dibasic acid metabolism (gmx00660) was enriched alone in the combination of *G. sinensis* with *G. australis*, and sphingolipid metabolism (gmx00600); histidine metabolism (gmx00340); and valine, leucine, and isoleucine biosynthesis (gmx00290) were individually enriched in the combination of *G. sinensis* and *G. microphylla*. All of the difference combinations were enriched in galactose metabolism (gmx00052) (Fig. 3.c).

According to the VIP value from large to small, the first 50 known metabolite IDs with obvious differences between pairs of varieties were extracted, and a total of 146 metabolites were screened out. After de-redundancy, a normalized heatmap analysis using the ComplexHeatmap R package showed that 15-methylpalmitate, 2-[(2Z)-4-hydroxy-3-(4-methylpent-3-en-1-yl)but-2-en-1-yl]benzene-1,3,5-triol, benzoquinoneacetic acid, and other metabolites (row cluster 2, column cluster 3) were highly expressed in *G. sinensis* and *G. fera*. In total, 35 metabolites (row cluster 3, column cluster 5), such as galabiose, D-maltose, raffinose, and sucrose, were highly expressed in *G. microphylla.* L-isoleucine, L-alloisoleucine, isovitexin 2”-O-(6″‘-feruloyl)glucoside, etc. (row cluster 4, column cluster 1) had higher expression levels in *G. australis*. Phlorizin, allose, 8-(1,2-dihydroxypropan-2-yl)-9-hydroxy-2H,8H,9H-furo[2,3−*h*]chromen-2-one, etc. (row cluster 5, column cluster 4) were highly expressed in *G. japonica* and *G. delavayi.*1,2-Di-(9Z,12Z,15Z-octadecatrienoyl)-3-(galactosyl-alpha-1-6-galactosyl-beta −1)-glycerol, dihydrocaffeic acid 3-O-glucuronide, verbenalin, etc. (row cluster 6, column cluster 1) were highly expressed in *G. velutina* (Fig. S1).

### 3.4 Nutrient element analysis among different *Gleditsia* species

**Fig. 4:**
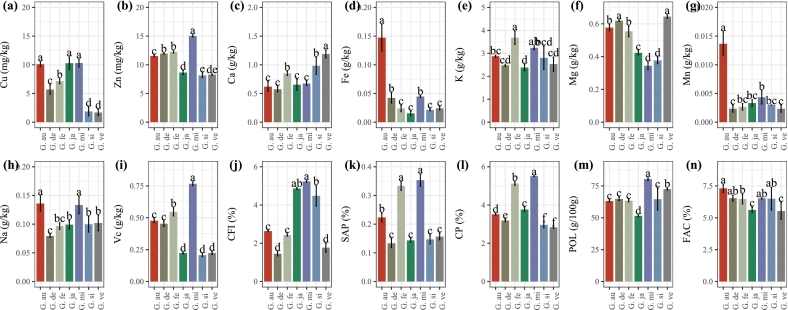
Distribution of nutrition and amino acid composition in the endoderm of *Gleditsia.****Note:*** a–n: Histogram corresponding to each indicator. CP: crude protein; CFI: crude fiber; FAC: fatty acid content; Vc: ascorbic acid content; POL: polysaccharide; SAP: saponins. In (a)–(n), data are shown as the mean ± SD (*n* = 3); different lowercase letters indicate significant differences at *p* < 0.05.

The nutritional composition, element distribution, and amino acid composition in *Gleditsia* ZJM were determined, and the results showed that the Cu content in the *Gleditsia* genus ranged from 1.69 to 10.3 mg/kg, *G. sinensis* (1.87 ± 0.77 mg/kg) and *G. velutina* (1.69 ± 0.53 mg/kg) had the lowest Cu content. The Cu contents for *G. australis*, *G. japonica*, and *G. microphylla* were comparable (Fig. 4.a). The Zn content ranged from 8.16 to 15.04 mg/kg (Fig. 4.b), and *G. microphylla* had the highest value of Zn (15.04 ± 0.2 mg/kg). The Ca content ranged from 0.57 to 1.19 g/kg (Fig. 4.c), *G. velutina* (1.19 ± 0.07 g/kg) had the highest Ca content. The Fe content ranged from 0.015 to 0.147 g/kg, with *G. australis* (0.15 ± 0.02 g/kg) having the highest Fe content in the *Gleditsia* genus (Fig. 4.d). The K content ranged from 2.38 to 3.68 g/kg (Fig. 4.e), and *G. fera* (3.68 ± 0.29 g/kg) had the highest content of K. The Mg content ranged from 0.34 to 0.64 g/kg (Fig. 4.f). The Mn content ranged from 0.002 to 0.013 g/kg (Fig. 4.g), and *G. australis* had the highest value of Mn. The Na content ranged from 0.079 to 0.136 g/kg (Fig. 4.h), and *G. australis* (0.136 ± 0.01 g/kg) and *G. microphylla* (0.133 ± 0.01 g/kg) had higher contents of Na. The Vc content ranged from 0.21 to 0.77 g/kg (Fig. 4.i), *G. microphylla* had the highest value of Vc (0.77 ± 0.01 g/kg); the CFI content ranged from 1.45 to 5.24% (Fig. 4.j), the SAP content ranged from 0.13 to 0.35% (Fig. 4.k); the CP content ranged from 2.83 to 5.51% (Fig. 4.l), meanwhile, *G. microphylla* had the highest CFI value (5.24 ± 0.04%), SAP value (0.35 ± 0.02%) and CP content (5.51 ± 0.02%); the POL content ranged from 51.49 to 80.37 g/100 g (Fig. 4.m), *G. japonica* had the lowest value of POL (51.49 ± 0.71 g/100 g); and the FAC content ranged from 5.53 to 7.31% (Fig. 4.n), *G. australis* had the highest value of FAC (7.31 ± 0.32%). A heatmap of the various amino acids' contents showed that *G. fera* had higher contents than other species in the *Gleditsia* genus (Fig. S2).

## 4 Discussion

Due to their extensive distribution and adaptability, plants of the *Gleditsia* genus are widely cultivated in the Guizhou, Henan, Shandong, Liaoning, and Yunnan provinces and in other areas in China. The endosperm of *Gleditsia*, known as ZJM, is rich in pectin and protein and is used as a thickener, stabilizer, and adhesive. The endosperm was frequently used as a traditional banquet sweet food with transparent color, smooth-tasting, low-fat, and high-carbohydrate (Liu et al., 2024). The seed coat of *G. sinensis* consisted of a palisade layer and light line that can hinder water entry into the seed (Zhu et al., 2021). The carbohydrates of the embryo and testa are of two classes: polysaccharides, not extractable by boiling water and oligosaccharides, soluble in 75% ethanol which, possibly, contain amino acids in their molecule (Navarro, Cerezo, & Stortz, 2002). Precipitation of the endosperm from the seed of *G. triacanthos* with 2-propanol yielded major amounts of galactomannan components, while the supernatant was mainly composed of arabinose-rich constituents (Navarro et al., 2002). The endosperm of *G. sinensis* also contains galactomannan, which has similar properties to plant products such as guar gum and fenugreek gum (Jiang, Zhang, Zhu, Liang, & Xiang, 2003). Mannan is the main component of hemicellulose, synthesized in the Golgi apparatus and then secreted into the cell wall through plasma membrane fusion vesicles (Northcote & Pickett-Heaps, 1966). It can be divided into four sub-families: linear mannans, glucomannan, galactomannan, and galactomannan (He et al., 2017). The synthesis of galactomannan is catalyzed by mannan synthase and α-galactosyltransferase (Reid, Edwards, Gidley, & Clark, 1992; Reid, Edwards, Gidley, & Clark, 1995). An aqueous solution of galactomannan is a pseudoplastic fluid, and the structure is a D-mannose backbone connected by β-1,4 bonds, with a D-galactose side chain connected to the backbone by α-1,6 glycosidic bonds. The macromolecules are in a state of an entangled network structure in nature (Jiang, Jian, Zhu, Zhang, & Sun, 2009; Jiang, Zhu, An, & Zhang, 2006). The viscosity of the solution is affected by the molecular weight of the galactomannan and the ratio of mannose to galactose (M/G) (Yantao Liu, Xu, Lei, Li, & Jiang, 2019). As a reserve of hemicellulose polysaccharide, galactomannan is stored in the cell walls of the endosperm and pericarp of the seed, retains water when the seed imbibes, and is used as a source of carbon and energy during germination (Joseph, Aravind, George, Varghese, & Sreelekha, 2013; Prajapati et al., 2013; Srivastava & Kapoor, 2005; Xiao-chun, Qun, & Jun-jie, 2008) Galactomannan is present in its highest amount in the seed 24 h after water absorption, and it is completely consumed within about 48 h after water absorption (Bakhshy, Zarinkamar, & Nazari, 2019). *G. microphylla* galactomannan is used in oil drilling, textile printing, dyeing, and animal feed, and GM-GM can be converted into manno-oligosaccharides (Xu et al., 2021). Seed extracts of *G. triacanthos* can be used not only as a source of galactomannan films suitable for the incorporation of antioxidant compounds for further application in the food industry but also as a source of the active compounds to be incorporated (Cerqueira, Souza, Martins, Teixeira, & Vicente, 2010). However, in previous research, the nutritional distribution and metabolic components of *Gleditsia* endosperm were not clear. In this study, endosperm from seven *Gleditsia* species native to China was used as the research object. Ripe pods from the seven species were collected. The endosperm moisture gradually decreases and the dry weight percentage of the three parts of the seeds (endosperm, hull, and embryo) remain constant during galactomannan deposition and maturation (Xu et al., 2020). In similar studies, 505 metaboliteswere found in three kinds of *Gleditsia* species (*G. delavayi*, *G. japonica, G. sinensis*) by UPLC–ESI–MS/MS (Lu, Ren, Zhao, Li, & Tan, 2024). In the study, the endosperm of the seven species of *Gleditsia* was obtained by means of manual and direct stripping and subjected to LC–MS/MS. A total of 4495 kinds of metabolites were detected, including 820 kinds of lipids and lipid-like molecules, 564 kinds of organic acids and their derivatives, and 431 kinds of organic oxygen compounds (Fig. 2.a). Identification and screening of metabolites through interspecific differences, 5-methylpalmitate,2-[(2Z)-4-hydroxy-3-(4-methylpent-3-en-1-yl)but-2-en-1-yl]benzene-1,3,5-triol, benzoquinoneacetic acid, and other metabolites (row cluster 2, column cluster 3) were highly expressed in *G. sinensis* and *G. fera*, galabiose, D-maltose, raffinose, and sucrose, were highly expressed in *G. microphylla.* Metabolites that are differentially expressed in different *Gleditsia* species can be used as markers for species identification.

A previous determination of the nutrient composition of individual species showed that the *G. delavayi* endosperm CP content was 2.37% ∼ 3.64%, the CFI content was under 1%, and the contents of mineral elements such as K, P, Ca, Mg, Na, Fe, Zn, and Mn were high (Weilin et al., 2017)*.* The polysaccharides of *G. delavayi* possessed certain antioxidant capacities in vitro, the IC (50) values for scavenging DPPH and ABTS+free radicals were 10.87 mg/mL and 6.67 mg/mL,respectively (Xu et al., 2023). In this study, the CP content in the *Gleditsia* genus ranged from 2.83 to 5.51% (Fig. 4.i), which is relatively consistent with previous research. The *Gleditsia* genus contains a large amount of polysaccharides—the content of POL ranged between 51.49 and 80.37 g/100 g, meanwhile, *G. microphylla* had the highest POL content (Fig. 4.m). In a prior study, rice was found to contain 12,000  mg/kg P and 8750  mg/kg K in the bran fraction and 1520  mg/kg P and 1230  mg/kg K in the resulting white rice fraction, while the P and K levels in the endosperm were 400–500  mg/kg and 600–700  mg/kg, respectively (Yao, Chen, & Sun, 2020). The nutrient element analysis results showed that the *Gleditsia* genus has a high content of K and a low content of Na, with a K/Na ratio ranging from 19.53% to 42.52%. The K levels were 2.232–3.863 g/kg, higher than those found in the endosperm of rice.

In the comparison of seed traits and endosperm metabolic components among *Gleditsia* species, *G. fera* had the fewest differences with 188 kinds when compared to *G. sinensis*, with the contents of a majority of amino acid indicators being higher than those in other species (Fig. S2). *G. fera* has been subjected to selection and there is a fast-growing cultivar available. Grafting onto *G. fera* as a rootstock increases the accumulation of photosynthetic products in the grafted plants (Xiao, Zhao, Wang, & Jian, 2023b). For growers whose harvest target is pod production, *G. fera* could be a good planting choice due to its fast growth, high seed yield, and full seeds.

## 5 Conclusions

This was the first comparative study of the metabolite composition and nutritional content of *Gleditsia* endosperms across species. A total of 4495 metabolites were identified, including 820 lipids and 564 organic acids and derivatives. *G. microphylla* had higher levels of Na, Vc, CFI, SAP, CP, and POL. The metabolites of *G. fera* showed the fewest differences from those of *G. sinensis*, and most of the amino acid contents of *G. fera* were higher than those of other species. Based on its fast growth, high seed yield, and excellent amino acid content, *G. fera* is a good choice for cultivation and promotion.

## Funding

This research was funded by the Characteristic Forestry Industry Research Project of Guizhou Province (GZMC-ZD20202102; GZMC-ZD20202098;2020-23), the China National Key R&D Program (Grant No. 2022YFD1601712-1), the Science and Technology Plan Project of Guizhou Province ([2022] general 102)

## CRediT authorship contribution statement

**Feng Xiao:** Formal analysis, Writing – original draft. **Yang Zhao:** Formal analysis, Funding acquisition. **Xiurong Wang:** Writing – review & editing. **Xueyan Jian:** Resources. **Fuhua Liu:** Resources.

## Declaration of competing interest

The authors declare that they have no known competing financial interests or personal relationships that could have appeared to influence the work reported in this paper.

## Data Availability

Data will be made available on request.

## Appendix A Supplementary data

Supplementary material Image 1
