# Supplementary material for: Metabolite components and nutritional composition of the endosperm in seven species from Gleditsia
Source: Food Chem X. 2024 Apr 4;22:101340. doi: 10.1016/j.fochx.2024.101340 (PMC11063355; doi:10.1016/j.fochx.2024.101340)
Supplement: Supplementary file 1 — Supplementary material [file mmc1.docx]

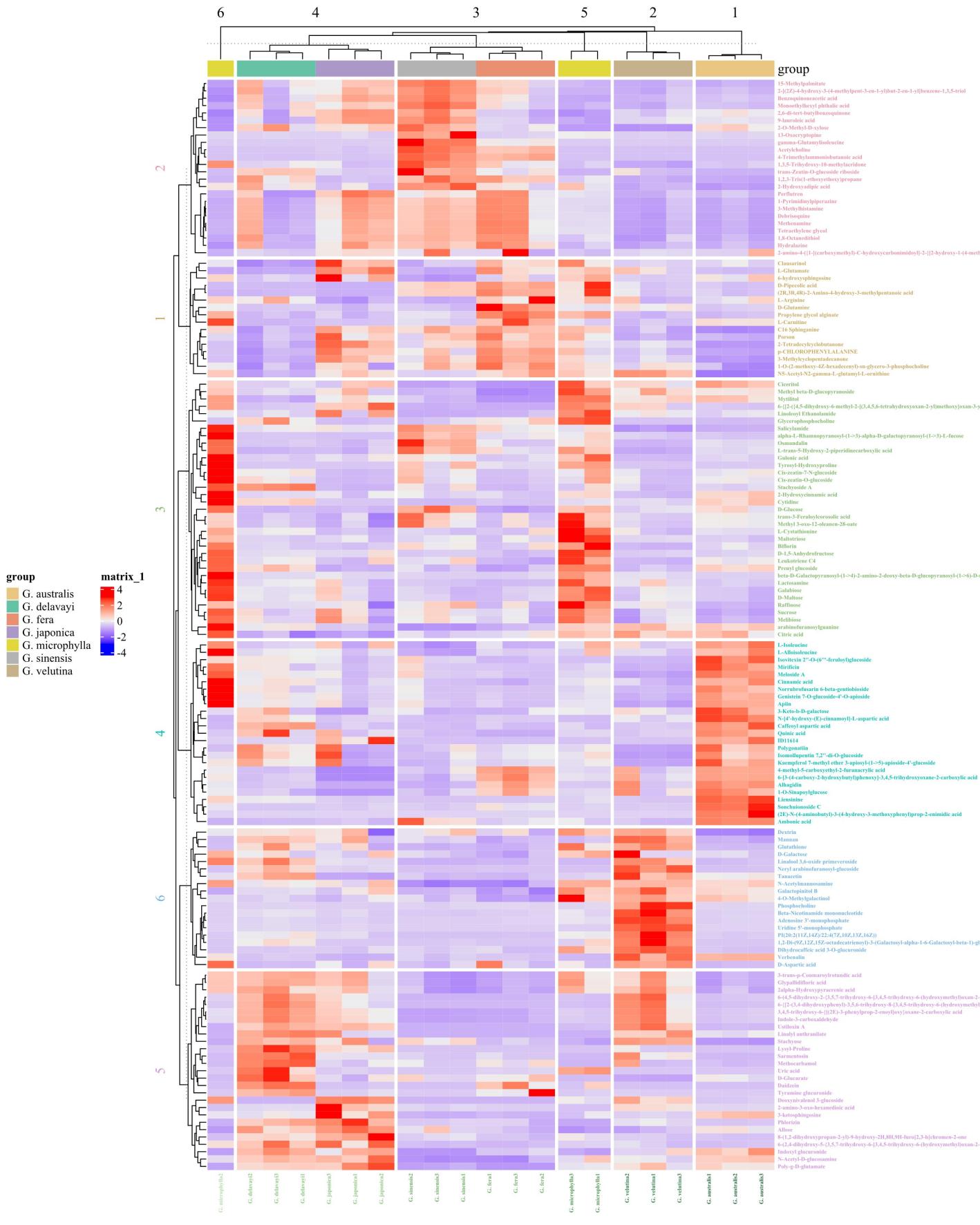


**Supplementary Figure 1.** Differential metabolite heatmap


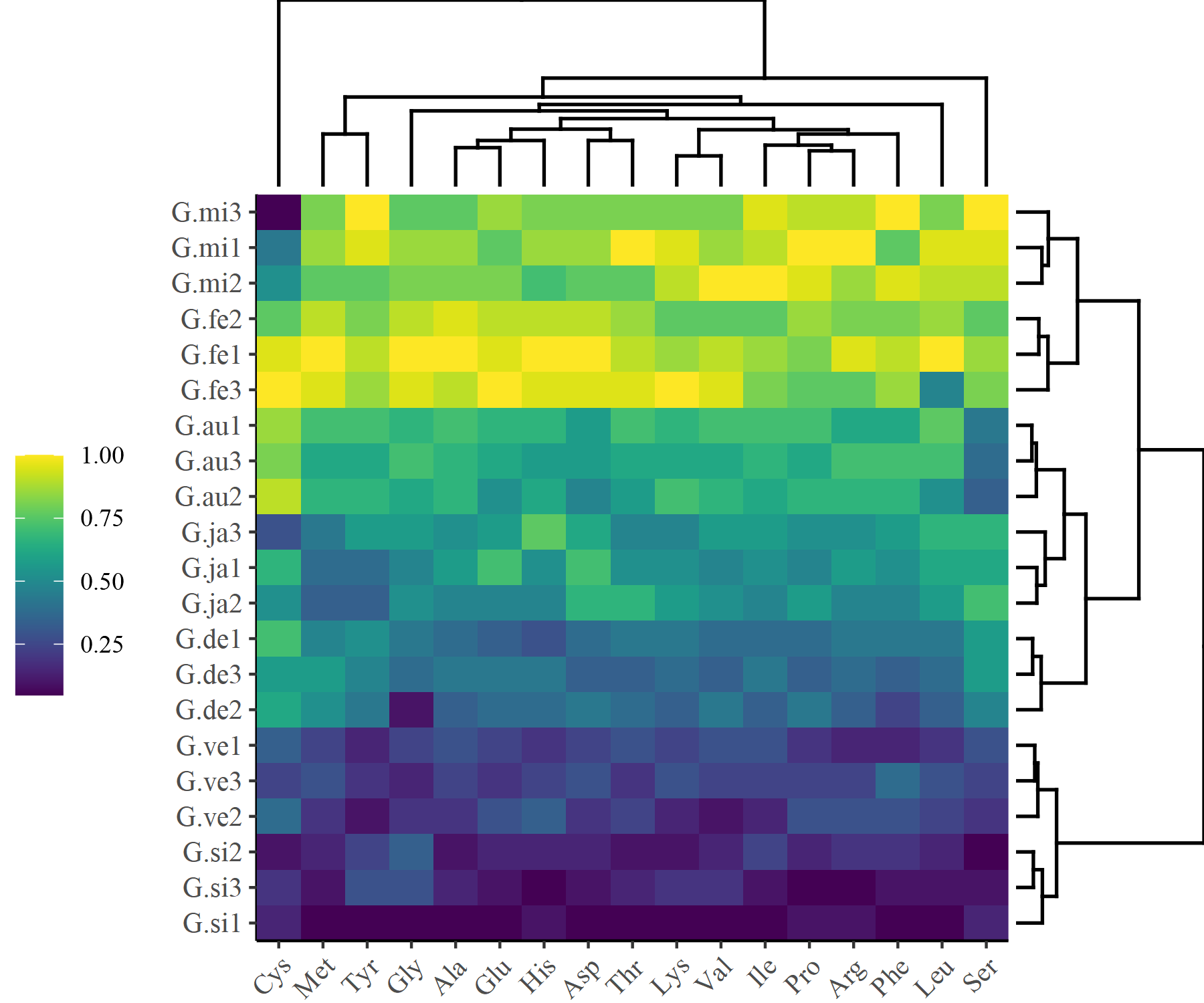


**Supplementary Figure 2.** Heatmap of different amino acid contents in the genus *Gleditsia.* Note: Asp: Asparticacid, Glu: Glutamicacid, Ser:Serine, His: Histidine, Gly: Glycine, Thr: Threonine, Arg: Arginine, Ala: Alanine, Tyr: Tyrosine, Cys: Cysteine, Val: Valine, Met: Methionine, Phe: Phenylalanine, Ile:Isoleucine, Leu:Leucine, Lys:Lysine, Pro: Proline.
